# Efficacy of Conservative Techniques for Mechanical Facial Rejuvenation: A Systematic Review

**DOI:** 10.1093/asjof/ojaf144

**Published:** 2025-11-04

**Authors:** Shaikh Sanjid Seraj, Onyedi Moses, Yasmin Kamel, Aljawhara S H Almishwat, Jemi C Maliyil, Mohamed Dalmar, Ryan Faderani, Afshin Mosahebi

## Abstract

A boom in the online popularity of conservative mechanical facial rejuvenation techniques, such as facial exercises, myofunctional therapy, and manual massages, has proliferated as noninvasive alternatives to aesthetic procedures. Despite this, scientific evidence regarding their efficacy and safety remains unclear. The aim of this study is to determine the efficacy of conservative mechanical facial exercises and related paraphernalia or adjuncts in achieving optimal facial aesthetic outcomes. A systematic review was conducted in accordance with the Preferred Reporting Items for Systematic Reviews and Meta-Analyses guidelines and registered on PROSPERO. Databases, including PubMed/MEDLINE, Embase, Web of Science, SciELO, AMED, PROQUEST, Google Scholar, CENTRAL, OVID, and SCOPUS, were searched for studies evaluating mechanical facial exercises in adults. Studies reporting objective and validated subjective facial aesthetic outcomes were included. Risk of bias was assessed using the Cochrane and JBI tools. Twelve studies comprising 321 women aged 30 to 70 met the inclusion criteria. Interventions included facial exercises (*n* = 6), myofunctional therapy (*n* = 4), and manual massage (*n* = 2). Reported improvements were confined to localized regions, such as the cheeks, jawline, and periorbital areas. Tools used included Cutometers (Courage + Khazaka electronic GmbH, Cologne, Germany), ultrasound, expert/self-assessments, and quality-of-life metrics. No adverse events were reported; however, methodological heterogeneity, small sample sizes, and a lack of male participants limited generalizability. Although some region-specific aesthetic improvements have been observed, current evidence remains insufficient to establish the efficacy of these mechanical facial rejuvenation techniques with confidence. The literature lacks standardized protocols that account for hormonal status and facial anatomy variability. Larger, randomized controlled trials with diverse populations are necessary to improve the current body of evidence.

**Level of Evidence:** 3 (Therapeutic)

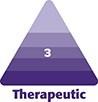

Conservative treatments targeting facial rejuvenation, verified by so-called “social media influencers” and advertised across aesthetic clinics and blogs, have emerged on social media platforms, including search engines (Figure 1A-C).^1^ In an effort to stay relevant, aesthetic providers often promote these beauty and aesthetic trends to enhance their marketing strategies, thereby fueling engagement, viewership, and, ultimately, sales.

**Figure 1.**
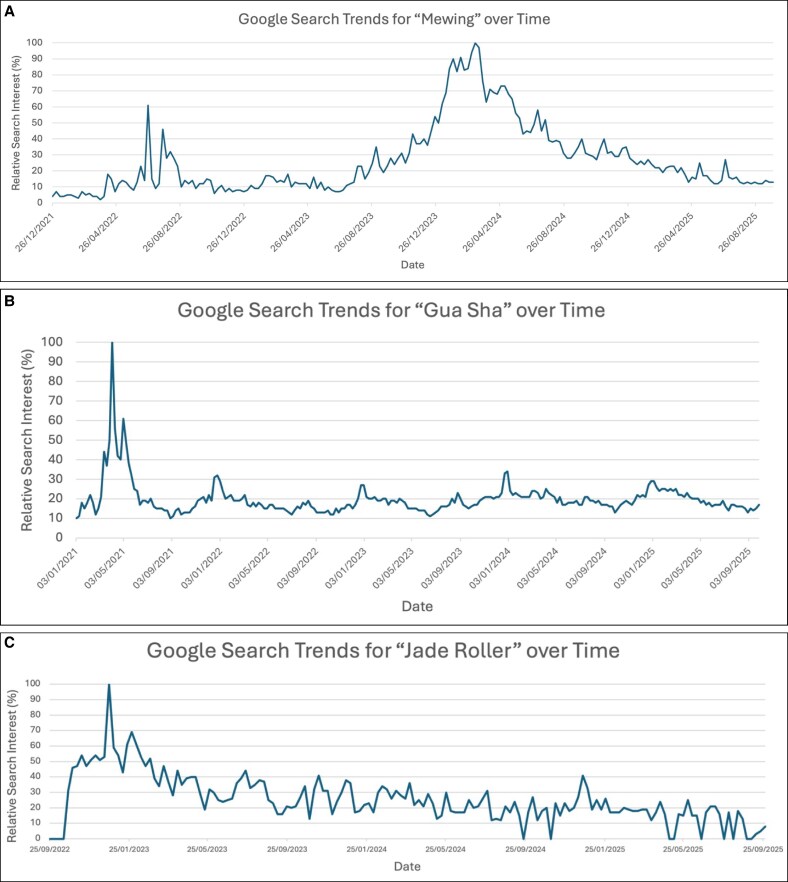
Google Trends Interest scores for “Mewing” (A; timeframe: 26/12/2021-26/08/2025); “Gua Sha” (B; timeframe: 03/01/2021-03/09/2025); and “Jade roller” (C; timeframe: 25/09/2022-25/09/2025).

Noninvasive mechanical facial rejuvenation techniques encompass a range of practices that involve facial exercises, self-massage tools, and structural positioning of the facial musculature. Among these, mewing, gua sha, and jade rollers have gained significant popularity as alternatives to more invasive aesthetic interventions (Figure 1A-C).^1^ The assumed benefits of such techniques provide users with a nonsurgical and low-cost option to achieve aesthetic outcomes that surgical or invasive nonsurgical interventions could otherwise achieve. As providers of aesthetic beauty, the aesthetic physician and plastic surgery community must understand these purported techniques to better inform patients and users of their efficacy based on the updated literature.

Mewing is a type of facial exercise that involves forced tongue posture against the hard and soft palate of the mouth.^2^ Purported claims include improvement in facial structure, jawline definition, and airway function over time. John Mew popularized the method, gaining traction because of anecdotal evidence and viral social media content.^2,3^ Proponents suggest long-term adherence can influence maxillofacial development and improve a defined jawline.^2^ However, there is a paucity across the literature evaluating the efficacy of mewing in achieving significant aesthetic or functional changes, often relying on anecdotal or low-level evidence.

“Gua sha” is a traditional Chinese medicine practice that involves skin scraping using a smooth-edged tool, often made of jade or rose quartz.^4^ Similarly, jade rollers are handheld massage tools traditionally used to enhance circulation and reduce facial tension perceptively.^5^ In the context of facial rejuvenation, both tools are believed to improve lymphatic drainage, puffiness, blood circulation, and minor skin benefits.^6^ Claims of significant long-term structural changes remain unproven in rigorous scientific studies.^7^ Despite this, its popularity has surged, particularly among individuals seeking noninvasive skin tightening and contouring methods.

Across the wider literature, facial aesthetic outcomes primarily rely on subjective and objective measures, including patient-reported satisfaction, clinician assessments, and aesthetic subunits.^8-10^ However, the literature lacks the efficacy of achieving optimum facial aesthetic outcomes using noninvasive mechanical facial rejuvenation techniques. Recent systematic reviews dating from a decade ago have examined this phenomenon, often concluding insufficient evidence based on the paucity and quality of literature and recommending larger-scale studies.^11-13^

This systematic review aimed to determine the efficacy of conservative mechanical facial exercises and related paraphernalia or adjuncts in achieving optimum facial aesthetic outcomes.

## METHODS

This systematic review was conducted in line with Preferred Reporting Items for Systematic Reviews and Meta-Analyses (PRISMA) guidelines.^14^ This review protocol was registered on PROSPERO^15^ (CRD42024619774).

As per the Cochrane guidelines, the search strategy was based on the patient, intervention, comparator, and outcome (PICO) framework.^16^

- Patient: adults (>18 years) who have undergone mechanical facial exercises in the context of facial aesthetic outcomes.
- Intervention: mechanical facial exercises, with or without adjuncts.
- Comparator: absence of mechanical facial exercises, with or without adjuncts.
- Outcome: pre- and postintervention findings, across both objective measuring tools and subjective validated scoring systems.

No publication date restrictions were used, and the search was restricted to articles published in English. A bespoke search strategy was devised, combining MeSH index and free-text terms relating to facial exercise, noninvasive tools, facial rejuvenation, and facial aesthetic outcomes (Appendix 1). The search strategy was applied to PubMed/MEDLINE, Embase, Web of Science, SciELO, AMED, PROQUEST, Google Scholar, Cochrane Central Register of Controlled Trials, and SCOPUS (Appendix 2). Reference lists of included studies were further explored for additional eligible studies. This study was compliant with the PRISMA guidelines (Appendix 3).^14^

Randomized controlled trials (RCTs), observational studies, cohort studies, and case–control studies involving both male and female patients were eligible for inclusion. Editorials, case reports, animal studies, letters, opinion pieces, systematic reviews, literature reviews, narrative reviews, conference abstracts without full texts, and studies examining surgical or invasive interventions in conjunction with mechanical facial exercises were excluded. Studies in pediatric patients (<18 years) were also excluded.

The ROB2 tool was used to determine the risk of bias for eligible RCTs.^17^ The JBI risk of bias tool was used to assess the risk of bias for nonrandomized controlled studies.^18^ Each study was analyzed for selection, performance, detection, and reporting biases.

Data extraction was performed by 2 independent reviewers (Y.K. and A.S.H.A.) using a predetermined electronic form (Google Sheets). The accuracy of the extracted data was ensured among the authors. The data included the study design, participant characteristics, intervention, comparator, outcomes measured, and results. A third independent reviewer (S.S.S.) was consulted to resolve any discrepancies in data extraction. Descriptive analysis was conducted because heterogeneity was consistent across the finalized studies.

## RESULTS

### Search Results

**Figure 2.**
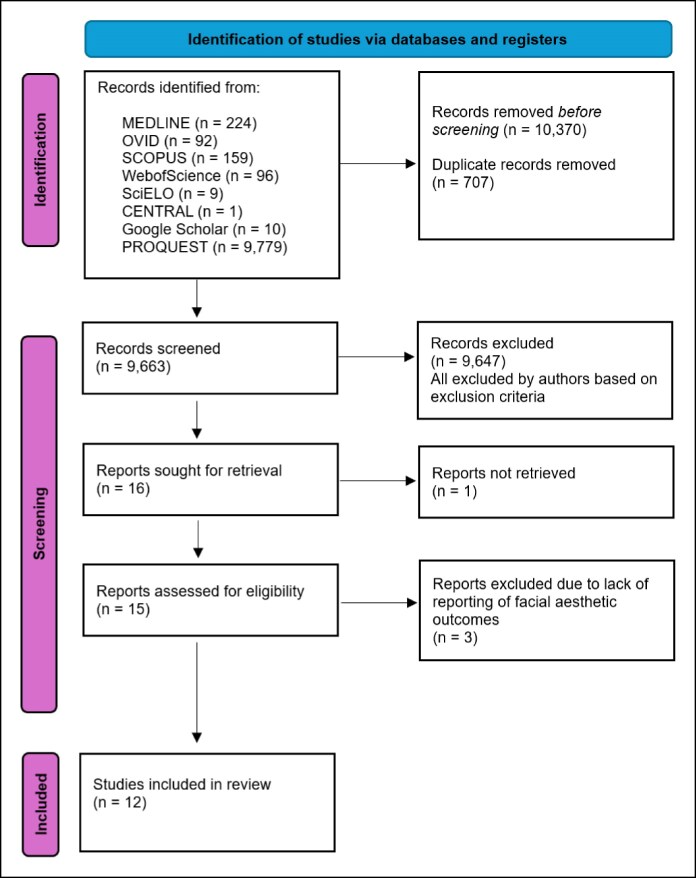
Preferred Reporting Items for Systematic Reviews and Meta-Analyses diagram of search results for the systematic review.

A total of 10,370 papers were identified across the following databases and registers: 224 from MEDLINE, 92 from OVID, 159 from SCOPUS, 96 from Web of Science, 9 from SciELO, 1 from CENTRAL, 10 from Google Scholar, and 9779 from PROQUEST. Initial searches were conducted on October 1, 2024. Seven hundred and seven duplicate papers were removed, and 9663 papers underwent title and abstract screening. The PRISMA diagram (Figure 2) summarizes the methodology, illustrating the screening process for the finalized papers. A final 15 papers were examined for full paper review. Following full paper reviews, 3 publications were excluded because they all lacked reporting of facial aesthetic outcomes. For this reason, only 12 articles met the inclusion criteria.

### Study Characteristics

Four pre-post observational studies, 2 non-RCTs, 5 RCTs, and 1 cross-sectional (quasi-experimental) study were identified (Supplemental Table 1). Five studies were conducted in Brazil, 3 in South Korea, and 1 in each of the United States, Belgium, Spain, and Malaysia. Five studies assessed facial exercise, 4 assessed myofunctional therapy, and 3 assessed manual therapy/craniofacial massage, with 2 studies assessing the use of an adjunct. Eight studies used a comparator group either as no intervention or use of an alternative treatment.

### Study Demographics

Across the included studies (Supplemental Table 1), all 321 participants (100%) were women aged between 30 and 70. Ninety-six participants underwent facial exercises, 25 participants underwent massage therapy, and 70 participants used external paraphernalia either as an orofacial device or a “Ki energy” device.

### Outcome Measures

#### Facial Exercises

Across the included studies, 5 of the 12 (Supplemental Table 2), involving 144 patients in total, investigated the efficacy of facial exercises on improving facial aesthetics, either as monotherapy or in combination.^19-23^ In 1 study, the authors used a mouthpiece with weighted, “dumbbell” like ends called a Pao device (MTG Co. Ltd, Nagoya, Japan).^21^ Interventions were deemed “facial exercises” if there was a deliberate, active contraction, and relaxation of facial muscles, with or without resistance, in repetitions. Exercise programs ranged between 7 and 20 weeks, and studies were clinically heterogeneous in methodology. Aesthetic improvement was measured by self or expert assessment (*n* = 4), skin elasticity readings with a Cutometer (Courage + Khazaka electronic GmbH, Cologne, Germany; *n* = 1), and facial muscle thickness and surface area with ultrasound (*n* = 1).^19-21-23^ Areas with significant aesthetic improvement (ie, *P* < .05 in the primary outcome measure across all facial exercise studies) included the skin over the cheeks and forehead (*n* = 2, 60 patients), midfacial and jawline surface distances (*n* = 1, 50 patients), zygomaticus and digastric muscle bulk (*n* = 1, 50 patients), and lower face and neck skin elasticity (*n* = 1, 16 patients).

#### Myofunctional Therapy

Four of 12 studies (Supplemental Table 2), involving 144 patients in total, assessed aesthetic changes associated with myofunctional therapy.^23-26^ One study used an oral rehabilitation device (Patakara LIP Trainer, Patakara, Punta Gorda, Spain). Interventions aimed at improving orofacial and stomatognathic function (speech, mastication, swallow, etc) were considered myofunctional therapy. As such, aesthetic change was either a primary (*n* = 2) or secondary (*n* = 2) outcome.^23-26^ Exercise programs ranged between 8 and 24 weeks, with lesser clinical heterogeneity than studies assessing facial exercises. Aesthetic improvement was measured by self or expert assessment (*n* = 3) and skin elasticity readings with a Cutometer (*n* = 1).^23-26^ Areas with significant improvement included forehead, glabellar, and periorbital wrinkles (*n* = 1, 44 patients) and bilateral lower face skin elasticity (*n* = 1, 13 patients). However, 2 studies, across 57 patients, found no significant aesthetic improvement.^24,25^

#### Manual Therapy and Massages

Three of the 12 studies (Supplemental Table 2), involving 93 patients in total, focused on manual therapy and/or massage techniques performed by participants (*n* = 2) or experts (*n* = 1).^27-29^ Interventions involving using an external force to move/stretch/vibrate facial muscles passively were included in this category. Studies lasted 3 to 15 weeks, with the most heterogeneity compared with other interventions. Aesthetic improvement was measured by self or expert assessment (*n* = 3), menopause symptoms and quality of life self-assessment (*n* = 2), and facial morphometric analysis software (*n* = 1).^27-29^ Areas of significant improvement included the general signs of aging (expert assessment; *n* = 1, 16 patients), body image/mental health perception (self-assessment; *n* = 1, 50 patients), paralateronasal line, mandibular projection, and nasolabial angle (facial morphometric analysis; *n* = 1, 27 patients).

#### General Trends

Five studies, across 167 patients, measured quality of life improvement (Supplemental Table 2), all revealing a significant improvement in self-perception and wellness following the intervention.^23,25,27-29^

### Risk of Bias

**Figure 3.**
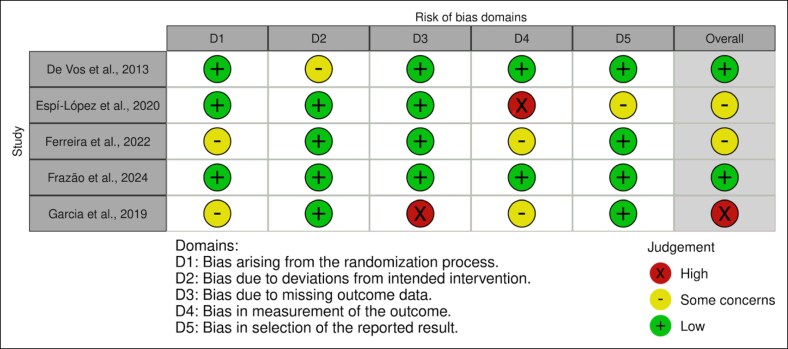
ROB2 tool for risk of bias assessment of randomized controlled trials included in the systematic review.

**Figure 4.**
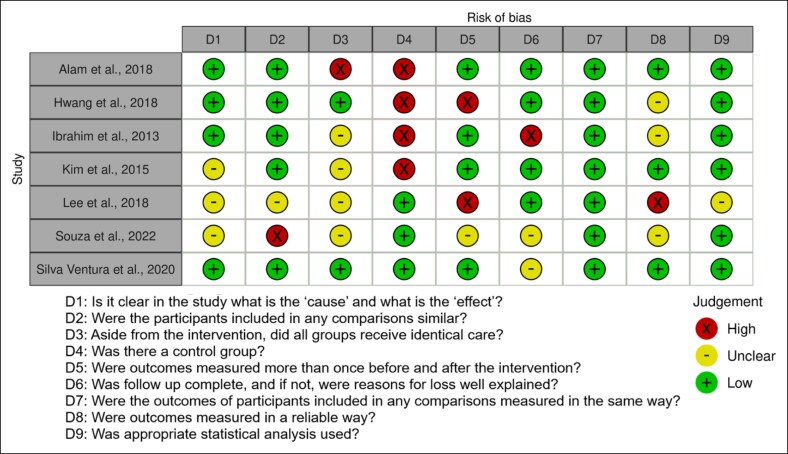
JBI tool for risk of bias assessment of nonrandomized controlled trials included in the systematic review.

Risk of bias analysis (Figures 3, 4) demonstrated an overall moderate bias level. The lack of control groups and lack of identical baseline treatment between groups were areas at greatest risk of bias. In contrast, appropriate measurement of outcomes and statistical analyses were areas at least risk of bias.

## DISCUSSION

This systematic review evaluated the efficacy of conservative mechanical techniques—primarily facial exercises, myofunctional therapy, and manual therapy—in improving facial aesthetics. Following full paper screening, 12 studies involving 321 participants were included. Despite increasing public interest and anecdotal popularity, the scientific literature remains limited, and the existing studies demonstrate considerable heterogeneity in design, intervention, and outcome assessment.^1^

The most prominent finding was the marked heterogeneity across the included studies. Finalized studies included a range of study designs: observational studies, non-RCTs, RCTs, and quasi-experimental studies. Intervention durations ranged between 3 and 24 weeks, and outcome measures varied substantially, including self-assessments, expert facial evaluations, ultrasound-based muscle assessments and Cutometer-based elasticity readings. Classifications have attempted to group areas of the face based on “facial units,” which could help further categorize and standardize aesthetic outcomes by regional anatomy.^8^ Given the marked heterogeneity, it is incumbent upon the aesthetic surgeon/practitioner community to help define a standardized set of outcomes when using these conservative mechanical facial techniques.

A notable limitation is the demographic homogeneity of the study populations. All participants across all 12 studies were women aged 30 to 70, with a strong bias toward postmenopausal cohorts. Although this demographic may reflect the primary consumer group for facial rejuvenation therapies, it introduces potential physiological biases.^30^ Postmenopausal hormonal shifts, particularly reductions in estrogen, have well-documented effects on skin elasticity, collagen content, subcutaneous fat distribution, and muscle tone.^31,32^ These variables may influence the baseline appearance and the responsiveness to mechanical interventions, making it unclear whether observed improvements would translate to younger or male populations. The complete absence of male participants in this study aligns with the wider literature, which also raises questions about gender-specific facial anatomy and muscle dynamics that warrant further investigation.^30^

Facial exercises, examined in 6 studies, showed statistically significant improvements in localized regions such as the cheeks, forehead, and jawline. Specific metrics included increased zygomaticus and digastric muscle bulk, improved cheek and midface contour, and enhanced lower facial skin elasticity. However, these improvements were reported in small, methodologically diverse studies.^19,20,22,24,27,28^ Notably, only 2 studies used objective tools such as ultrasound or the Cutometer to measure outcomes, with the remainder relying on subjective assessments.^21,26^ Even where statistical significance was demonstrated, translating this into clinical improvements remains unclear. Small percentage increments of facial muscle bulk may not be recognized by both patients and clinicians as meaningful facial rejuvenation.

Myofunctional therapy studies demonstrated benefits in the periorbital, glabellar, and lower face regions but similarly suffered from inconsistent outcome reporting. Although even more heterogeneous, manual therapies suggested possible benefits in facial contouring, self-perception, and quality of life.^21,26,29,33^ Still, the evidence was again limited by small sample sizes and a lack of blinding or controls.

Although most studies reported some degree of aesthetic or psychological improvement, these findings must be interpreted cautiously, with only a few studies confined to some anatomical regions.^20,21,27,28,33^ Moreover, the lack of long-term follow-up in most studies hinders the assessment of the prognosis of these noninvasive interventions.

Most researchers in the included studies were nonclinicians, such as biochemists, physiotherapists, and bioengineers. With only 3 of the 12 studies involving medical doctors, none included input from surgeons or plastic surgeons. Given the subjective nature of what constitutes “facial rejuvenation,” it is essential that future studies are led by clinicians and members of the aesthetic community, who are best positioned to perform these aesthetic assessments. Within the wider literature, clinicians have attempted to standardize facial aesthetic outcomes.^8,34-36^ This key methodological gap in expert-led research, therefore, necessitates further higher-quality studies performed by professionals within the aesthetic community.

The studies included in this systematic review were published in journals with a mean impact factor of 1.18, indicating limited academic reach and modest citation impact. This stark disparity highlights the significant disconnect between the media's enthusiasm for conservative facial rejuvenation techniques and scientific rigor, as well as clinical evidence-based exposure. With such a low-impact dissemination, it raises concerns surrounding the sources that inform public perception and the claims made by private clinics and medspas.

In this systematic review, no complications were reported, further reinforcing the favorable safety profile of conservative facial rejuvenation techniques. Often marketed as “natural” alternatives to invasive aesthetic procedures, their popularity centers around their perceived safety, affordability, and accessibility. This concurs with previous systematic reviews on conservative mechanical facial techniques, which report them as safe to use.^37^ Anecdotal evidence often outpaces rigorous clinical research, creating a gap between public perception and scientific consensus.^38^

This systematic review is not without limitations. Firstly, the included studies were methodologically heterogeneous, with small sample sizes of a narrow demographic cohort of women aged 30 to 70. Secondly, despite the comprehensive multi-database search strategies, the rapid proliferation of facial exercises and adjuncts across social media means published terminology may not fully capture the whole scope of practices being used for conservative mechanical facial rejuvenation. The evolving nomenclature surrounding facial exercises and adjuncts presents an inherent challenge to systematic searching. Thirdly, many studies were conducted by nonclinicians in low-impact factor journals, which raises concerns over methodological rigor and dissemination. Lastly, the lack of long-term follow-up limits conclusions surrounding long-term efficacy. These limitations necessitate cautious interpretation of the findings, requiring further clinically relevant robust studies.

Future research investigating the efficacy and safety of conservative mechanical facial rejuvenation techniques must further consider the following suggestions. Firstly, standardized protocols with clear intervention regimes and validated patient outcome measures, both using patient-reported outcomes measures and objective measurement tools, must be examined. Secondly, including diverse cohorts from various ethnic backgrounds, ages, and genders will enhance the study's generalizability and further investigate outcomes arising from anatomical and physiological variations. Finally, higher-level studies, such as RCTs with long-term follow-up, are necessary to establish both short- and long-term efficacy.

## CONCLUSIONS

This systematic review assessed the current literature on the efficacy and safety of conservative mechanical facial exercises and adjunctive devices in improving facial aesthetic outcomes. Several studies have demonstrated improvements in muscle tone, skin elasticity, and subjective aesthetic evaluations. However, this systematic review is limited by heterogeneity, small sample sizes, and a paucity of standardized outcome measures. The interventions reviewed were consistently safe, and their increasing popularity likely reflects their affordability, accessibility, and noninvasive nature rather than robust clinical evidence to justify these conservative mechanical techniques for effective facial rejuvenation.

## Supplementary Material

ojaf144_Supplementary_Data
