# Supplementary material for: Efficacy of Conservative Techniques for Mechanical Facial Rejuvenation: A Systematic Review
Source: Aesthet Surg J Open Forum. 2025 Nov 4;7:ojaf144. doi: 10.1093/asjof/ojaf144 (PMC12658320; doi:10.1093/asjof/ojaf144)
Supplement: ojaf144_Supplementary_Data [file ojaf144_supplementary_data.zip › Appendix 1.docx]

**Appendix 1**

Search strategy (main terms)

Myofunctional therapy [Mesh]

Myofunctional oral therapy

Orofacial myofunctional therapy

Oral myology

Orthotropics

Simoning

Facial exercise

Muscle retraining

Smile exercise

Lip exercise

Masseter exercise

Mandibular exercise

Face yoga

Lip strength exercises

Facial yoga

Mewing

Skin aging [Mesh]

Skin tightening

Wrinkles

Skin elasticity

aesthetic

Facial rejuvenation

Biting ball

Masseter ball

Clenching ball

Jade roller

Palatal expander

Gua sha
