# Supplementary material for: Efficacy of Conservative Techniques for Mechanical Facial Rejuvenation: A Systematic Review
Source: Aesthet Surg J Open Forum. 2025 Nov 4;7:ojaf144. doi: 10.1093/asjof/ojaf144 (PMC12658320; doi:10.1093/asjof/ojaf144)
Supplement: ojaf144_Supplementary_Data [file ojaf144_supplementary_data.zip › Appendix 2.docx]

**Appendix 2**

MEDLINE – 244 RESULTS

(((((Skin aging [Mesh]) OR (Skin tightening)) OR (Wrinkles)) OR (Skin elasticity)) OR (aesthetic)) OR (Facial rejuvenation)

AND

(((((((((((((((((((((Myofunctional therapy [Mesh]) OR (Myofunctional oral therapy)) OR (Orofacial myofunctional therapy)) OR (Oral myology)) OR (Orthotropics)) OR (Simoning)) OR (Facial exercise)) OR (Muscle retraining)) OR (Smile exercise)) OR (Lip exercise)) OR (Masseter exercise)) OR (Mandibular exercise)) OR (Face yoga)) OR (Lip strength exercises)) OR (Facial yoga)) OR (Mewing)) OR (Biting ball)) OR (Masseter ball)) OR (Clenching ball)) OR (Jade roller)) OR (Gua sha)

OVID – 92 RESULTS

(((((Skin aging [Mesh]) OR (Skin tightening)) OR (Wrinkles)) OR (Skin elasticity)) OR (aesthetic)) OR (Facial rejuvenation)

AND

(((((((((((((((((((((Myofunctional therapy [Mesh]) OR (Myofunctional oral therapy)) OR (Orofacial myofunctional therapy)) OR (Oral myology)) OR (Orthotropics)) OR (Simoning)) OR (Facial exercise)) OR (Muscle retraining)) OR (Smile exercise)) OR (Lip exercise)) OR (Masseter exercise)) OR (Mandibular exercise)) OR (Face yoga)) OR (Lip strength exercises)) OR (Facial yoga)) OR (Mewing)) OR (Biting ball)) OR (Masseter ball)) OR (Clenching ball)) OR (Jade roller)) OR (Gua sha)

SCOPUS – 159 RESULTS

(((((Skin aging [Mesh]) OR (Skin tightening)) OR (Wrinkles)) OR (Skin elasticity)) OR (aesthetic)) OR (Facial rejuvenation)

AND

(((((((((((((((((((((Myofunctional therapy [Mesh]) OR (Myofunctional oral therapy)) OR (Orofacial myofunctional therapy)) OR (Oral myology)) OR (Orthotropics)) OR (Simoning)) OR (Facial exercise)) OR (Muscle retraining)) OR (Smile exercise)) OR (Lip exercise)) OR (Masseter exercise)) OR (Mandibular exercise)) OR (Face yoga)) OR (Lip strength exercises)) OR (Facial yoga)) OR (Mewing)) OR (Biting ball)) OR (Masseter ball)) OR (Clenching ball)) OR (Jade roller)) OR (Gua sha)

Web Of Science – 96 RESULTS

(((((Skin aging [Mesh]) OR (Skin tightening)) OR (Wrinkles)) OR (Skin elasticity)) OR (aesthetic)) OR (Facial rejuvenation)

AND

(((((((((((((((((((((Myofunctional therapy [Mesh]) OR (Myofunctional oral therapy)) OR (Orofacial myofunctional therapy)) OR (Oral myology)) OR (Orthotropics)) OR (Simoning)) OR (Facial exercise)) OR (Muscle retraining)) OR (Smile exercise)) OR (Lip exercise)) OR (Masseter exercise)) OR (Mandibular exercise)) OR (Face yoga)) OR (Lip strength exercises)) OR (Facial yoga)) OR (Mewing)) OR (Biting ball)) OR (Masseter ball)) OR (Clenching ball)) OR (Jade roller)) OR (Gua sha)

SciELO – 9 RESULTS

(Skin aging) OR (Skin tightening)) OR (Wrinkles)) OR (Skin elasticity)) OR (aesthetic)) OR (Facial rejuvenation)

AND

(Myofunctional therapy) OR (Myofunctional oral therapy)) OR (Orofacial myofunctional therapy)) OR (Oral myology)) OR (Orthotropics)) OR (Simoning)) OR (Facial exercise)) OR (Muscle retraining)) OR (Smile exercise)) OR (Lip exercise)) OR (Masseter exercise)) OR (Mandibular exercise)) OR (Face yoga)) OR (Lip strength exercises)) OR (Facial yoga)) OR (Mewing)) OR (Biting ball)) OR (Masseter ball)) OR (Clenching ball)) OR (Jade roller)) OR (Gua sha)

CENTRAL – 1 RESULTS

GOOGLE SCHOLAR – 10 RESULTS

((Skin aging) OR (Skin tightening) OR (Wrinkles) OR (Skin elasticity) OR (aesthetic) OR (Facial rejuvenation)) AND ((Myofunctional therapy) OR (Myofunctional oral therapy) OR (Orofacial myofunctional therapy) OR (Oral myology) OR (Orthotropics) OR (Simoning) OR (Facial exercise) OR (Muscle retraining) OR (Smile exercise) OR (Lip exercise) OR (Masseter exercise) OR (Mandibular exercise) OR (Face yoga) OR (Lip strength exercises) OR (Facial yoga) OR (Mewing) OR (Biting ball) OR (Masseter ball) OR (Clenching ball) OR (Jade roller) OR (Gua sha))

AMED – 1 RESULTS

((Skin aging) OR (Skin tightening) OR (Wrinkles) OR (Skin elasticity) OR (aesthetic) OR (Facial rejuvenation)) AND ((Myofunctional therapy) OR (Myofunctional oral therapy) OR (Orofacial myofunctional therapy) OR (Oral myology) OR (Orthotropics) OR (Simoning) OR (Facial exercise) OR (Muscle retraining) OR (Smile exercise) OR (Lip exercise) OR (Masseter exercise) OR (Mandibular exercise) OR (Face yoga) OR (Lip strength exercises) OR (Facial yoga) OR (Mewing) OR (Biting ball) OR (Masseter ball) OR (Clenching ball) OR (Jade roller) OR (Gua sha))

PROQUEST – 10,346 RESULTS

((Skin aging) OR (Skin tightening) OR (Wrinkles) OR (Skin elasticity) OR (aesthetic) OR (Facial rejuvenation)) AND ((Myofunctional therapy) OR (Myofunctional oral therapy) OR (Orofacial myofunctional therapy) OR (Oral myology) OR (Orthotropics) OR (Simoning) OR (Facial exercise) OR (Muscle retraining) OR (Smile exercise) OR (Lip exercise) OR (Masseter exercise) OR (Mandibular exercise) OR (Face yoga) OR (Lip strength exercises) OR (Facial yoga) OR (Mewing) OR (Biting ball) OR (Masseter ball) OR (Clenching ball) OR (Jade roller) OR (Gua sha)) AND stype.exact("Scholarly Journals") AND la.exact("English") AND (at.exact("Feature" OR "Article" OR "Evidence Based Healthcare") AND la.exact("ENG") AND pd(20141002-20241002) AND PEER(yes))
