# Supplementary material for: Efficacy of Conservative Techniques for Mechanical Facial Rejuvenation: A Systematic Review
Source: Aesthet Surg J Open Forum. 2025 Nov 4;7:ojaf144. doi: 10.1093/asjof/ojaf144 (PMC12658320; doi:10.1093/asjof/ojaf144)
Supplement: ojaf144_Supplementary_Data [file ojaf144_supplementary_data.zip › Supplementary_Table_1_final.docx]

| Author, Year | Country | Impact Factor | Researcher background (by department) | Number of participants  (% of female participants) | Age range | Study design | Comparator | Intervention |
| --- | --- | --- | --- | --- | --- | --- | --- | --- |
| Alam et al., 2018 | USA | 7.99 | Dermatology  /ENT  /Skin surgery  (all doctors) | 16 (100%) | 40 – 65 | Pre-post observational study | No | Facial exercises under resistance |
| De Vos et al., 2013 | Belgium | 1.219 | Dermatology (doctor)  /Speech & language pathology | 18 (100%) | 40 – 60 | RCT | Yes | Experimental group: Facial exercises under resistance  Control group: No intervention |
| Espí-López et al., 2020 | Spain | 0.58 | Physiotherapist  /Psychologist  (non-clinical) | 50 (100%) | 45 – 65 | RCT | Yes | Experimental group: Craniofacial massage  Control group: No intervention |
| Ferreira et al., 2022 | Brazil | Not found | Speech & language  (non-clinical) | 27 (100%) | 30 – 78 | RCT | Yes | Experimental group 1: Suprahyoid muscle exercise  Experimental group 2: Suprahyoid muscle exercise and functional swallowing training  Control group: None |
| Frazão et al., 2024 | Brazil | 0.224 | Speech & language therapy  (non-clinical) | 30 (100%) | 50 – 60 | RCT | Yes | Experimental and Control groups: Orofacial Myofunctional Therapy Program  Experimental group only: EMG feedback |
| Garcia et al., 2019 | Brazil | Not found | Physiotherapy  (non-clinical) | 16 (100%) | 40 – 50 | RCT | Yes | Experimental and control groups: Lecture-based education on skin-care, diet, and general wellness.  Experimental group only: MT |
| Hwang et al., 2018 | South Korea | 2.235 | Physiotherapy  (non-clinical) | 50 (100%) | 30 – 63 | Pre-post observational study | No | Use of Pao device (facial exercise device) |
| Ibrahim et al., 2013 | Malaysia | 0.174 | Biomedical engineering  /Biochemistry  (non-clinical) | 13 (100%) | Mean age 44.7 +/- 3.4 SD. Range unspecified | Pre-post observational study | No | Orofacial myofunctional exercises with oral rehabilitation device (Patakara® LIP Trainer) |
| Kim et al., 2011 | South Korea | Not found | Biotechnology  (non-clinical) | 16 (100%) | 35 – 58 | Pre-post observational study | No | Facial exercises under resistance / KFRP |
| Lee et al., 2018 | South Korea | 0.675 | Alternative/  Complementary therapy  (Non-clinical) | 14 (100%) | Mean age 21.6. Range unspecified. | Cross-sectional study (Quasi-experimental) | Yes | Group 1: Alternative treatment SUKI  Group 2: TENS |
| Souza et al., 2022 | Brazil | 1.1 | Speech & language therapy  /Medical doctor | 44 (100%) | 50 – 65 | NRSI | Yes | Both groups: Aesthetic myofunctional therapy. |
| Silva Ventura et al., 2020 | Brazil | Not found | Physiotherapy | 27 (100%) | >40 (range unspecified) | NRSI | Yes | Group 1: MT  Group 2: MT and firming cream |

**Table 1:** Study Demographics

Key: ENT: Ear, Nose & Throat clinician/otolaryngologist, RCT: Randomised controlled trial, NRSI: Non-randomised controlled trial, MT: manual therapy, KFRP: Kyunghee Facial Resistance Program, SUKI: Superficial Using Ki energy Instruments, TENS: Transcutaneous Electrical Nerve Stimulation.
