# Supplementary material for: Efficacy of Conservative Techniques for Mechanical Facial Rejuvenation: A Systematic Review
Source: Aesthet Surg J Open Forum. 2025 Nov 4;7:ojaf144. doi: 10.1093/asjof/ojaf144 (PMC12658320; doi:10.1093/asjof/ojaf144)
Supplement: ojaf144_Supplementary_Data [file ojaf144_supplementary_data.zip › Supplementary_Table_2_final.docx]

| Author, Year | Outcome Measures | Relevant Results and Significant Findings | | | |
| --- | --- | --- | --- | --- | --- |
|  |  | Before | After | P value | Areas of Aesthetic Improvement |
| Alam et al., 2018 | Before and after signs of facial aging.  Validated MCFAP score. | 1.8 ± 0.7  1.6 ± 0.9 | 1.1 ± 0.6  0.9 ± 0.7 | p = 0.003  p = 0.003 | Improvement in upper and lower cheek fullness |
| De Vos et al., 2013 | Before and after signs of facial aging.  Scored using Visual Analogue Scale by non-experts and participants. | 41/49 | 60/30 | p = 0.004 | Improvement in upper lip when judged by external evaluators. |
| Espí-López et al., 2020 | RM-ANOVA’s estimated marginal mean for the SF-36, QSCPGSe score and MRS score | 17.77 ± 0.59 | 19.49, ± 0.49 | p < 0.05 | Improvement in mental health score. |
| Ferreira et al., 2022 | Before and after visual self-perception, muscle strength self-perception, and electromyography. | Group 1:  69.30%  Group 2: 78.81%  Control:  100.13% | Group 1:  105.58%  Group 2: 114.95%  Control:  117.64% | Group 1:  p = 0.011  Group 2: p = 0.021  Control:  p = 0.260 | Improvement in muscle activation measured by EMG, however no significant changes in aesthetic outcome associated with this. |
| Frazão et al., 2024 | Validated FASA score. 4 assessments of facial aging throughout study. | Assessment 1: 20.80 ± 4.60  Assessment 2: 21.87 ± 5.01 | Assessment 1: 18.33 ± 6.58  Assessment 2: 20.33 ± 6.13 | p < 0.05  p < 0.05 | Improvement in global signs of facial aging for a limited time – by the study endpoint (assessment 4), these changes had regressed to non-significant levels. |
| Garcia et al., 2019 | Before and after signs of aging/wrinkles.  Expert scoring via Facial Assessment Protocol  (validated by Micussi et.al). | Upper third of face:  01.8 ± 0.6  Middle third of face:  02.2 ± 0.7  Lower third of face:  02.0 ± 0.7 | Upper third of face:  01.2 ± 0.6  Middle third of face:  01.8 ± 0.8  Lower third of face:  01.7 ± 0.7 | Upper third of face:  p < 0.0001  Middle third of face:  p = 0.0141  Lower third of face:  p = 0.0129 | MT significantly decreased signs of aging/wrinkle compared to control.  Both MT and control groups satisfied with outcome. |
| Hwang et al., 2018 | Before and after quantitative measurement of facial muscle thickness, facial surface distances, and surface area of muscles of facial expression with ultrasound.  WSRS and FVS scales. | FVS wrinkles: 3.855 ± 1.715  FVS jawline:  4.000 ± 2.082 | FVS wrinkles: 5.130 ± 1.493  FVS jawline:  5.407 ± 1.666 | FVS wrinkles: p < 0.001  FVS jawline: p < 0.001 | Quantitative changes in facial muscle structure.  Significant difference in WSRS and Face Visual Scale scores pre and post intervention. |
| Ibrahim et al., 2013 | Before and after quantitative measurement of skin elasticity with Cutometer.  Labial closure and tongue elevation strength measured with an Oral Performance Instrument. | Right skin elasticity: 0.43 ± 0.16  Left skin elasticity: 0.48 ± 0.14 | Right skin elasticity: 0.57 ± 0.06  Left skin elasticity: 0.58 ± 0.05 | Right skin elasticity: p < 0.05  Left skin elasticity: p < 0.05 | Significant change in skin elasticity.  Improvement in orofacial myofunctional strength. |
| Kim et al., 2011 | Before and after quantitative measurement of skin elasticity with Cutometer. | 0.566 ± 0.025 | 0.779 ± 0.021 | p < 0.01 | Significant change in skin elasticity. |
| Lee et al., 2018 | Before and after buccinator tonus measured with myotonometer | - | - |  | Improvement in muscle tonus in both treatment groups. No significant difference between groups |
| Souza et al., 2022 | Before and after aesthetic myofunctional therapy. Evaluation with non-validated aesthetic assessment protocol found in literature.  SF-36 | Relevant values unspecified | Relevant values unspecified | Relevant values unspecified | Improvement in forehead, glabellar, and periorbital wrinkles.  Reduced muscle tension on forehead, glabella, eyes, lips, and cheeks. |
| Silva Ventura et al., 2020 | Before and after facial morphometric analysis with Facial Analysis Software (Dolphin Imaging version  12.0), patient satisfaction questionnaire, and validated GAIS. | - | - | Bilateral lateral mandibular projection between groups:  p = 0.04 | Improvement in paralateronasal line, mandibular projection, and nasolabial angle in both groups.  Significant change in mandibular projection between groups |

**Table 2:** Study Outcomes and significant findings

Key: Global Aesthetic Improvement Scale (GAIS), 36-Item Short Form Survey (SF-36), Non-validated Face Visual Scale (FVS), Validated Wrinkle Severity Rating Scale (WSRS), Facial Aging Signs Analysis (FASA), Menopause Rating Scale (MRS score), Body Satisfaction and Global Self Perception questionnaire (QSCPGSe score) and Merz-Carruthers Facial Aging Photoscales (MCFAP), MT: Manual therapy.
